# Increasing Frequency of Blackwater Fever in Ugandan Children Hospitalized with Severe Malaria, 2008–2025

**DOI:** 10.4269/ajtmh.26-0419

**Published:** 2026-10-27

**Authors:** Ruth Namazzi, Michael T. Hawkes, Kevin C. Kain, Robert O. Opoka, Anthony Batte, Chandy C. John, Andrea L. Conroy

**Affiliations:** ^1^ Paediatrics and Child Health, Makerere University College of Health Sciences, Kampala, Uganda;; ^2^ Global Health Uganda, Kampala, Uganda;; ^3^ Department of Pediatrics, University of British Columbia, Vancouver, Canada;; ^4^ University Health Network–Toronto General Hospital Department of Medicine, University of Toronto, Toronto, Canada;; ^5^ Sandra Rotman Centre for Global Health, Toronto, Canada;; ^6^ Centre for Child Health and Development, Makerere University College of Health Sciences, Kampala, Uganda;; ^7^ Department of Pediatrics, Indiana University School of Medicine, Indianapolis, Indiana, USA

## Abstract

Blackwater fever (BWF), which is characterized by dark urine associated with intravascular hemolysis during malaria infection, is increasingly reported among African children hospitalized with severe malaria. To examine temporal trends beyond previously recognized high-frequency districts in eastern Uganda, we analyzed longitudinal data from 3,340 children enrolled in five severe malaria studies conducted from 2008 to 2025 at Mulago National Referral Hospital in central Uganda and Jinja Regional Referral Hospital in southeastern Uganda. Blackwater fever was defined consistently as caregiver-reported passage of dark or cola-colored urine before admission. The frequency of BWF at admission increased from 24 of 150 children (16%) before 2010 to 259 of 489 children (53%) after 2020. In logistic regression models, calendar year remained associated with BWF after adjustment for demographic, clinical, and treatment-related factors (year-over-year odds ratio, 1.18; 95% CI, 1.03–1.34; *P* = 0.010). These findings support BWF as an emerging severe malaria complication requiring further investigation.

## INTRODUCTION

Blackwater fever (BWF), characterized by dark urine associated with intravascular hemolysis during malaria infection, is increasingly recognized among African children hospitalized with severe malaria. Blackwater fever is clinically significant because it is associated with severe anemia, acute kidney injury (AKI), persistent kidney disease, recurrent admissions, and postdischarge mortality.^1^ Although historically, BWF was typically an isolated event associated with quinine exposure among nonimmune adults,^2^ recent reports from Uganda describe recurrent disease episodes in children,^3^ suggesting potentially substantial long-term consequences for child health, survival, and health care systems.

Beginning in the late 2000s, clinicians in eastern Uganda observed increasing numbers of children admitted with severe malaria and dark urine suggestive of intravascular hemolysis with marked geographic variability in reported BWF frequency.^4^ Multiple reports of children presenting with dark urine syndrome in eastern Uganda prompted district-level epidemiologic investigations beginning in 2018, including a field investigation in Manafwa District. Medical record review (2013–2018) combined with active community case finding noted a sharp rise in incidence starting in 2015.^5^ Between 2019 and 2021, public health authorities in eastern Uganda initiated an investigation into the increasing reports of dark urine, reflecting growing concern about the rising incidence in the region.^6^ Although BWF commonly occurred in children with malaria, no additional triggers were identified.^5^^,^^6^ These investigations increased clinical awareness of BWF, enhanced documentation of dark urine, and resulted in community case-finding activities to better characterize disease occurrence. Whether BWF is increasing beyond previously recognized high-incidence regions and over longer time periods remains unclear. We, therefore, examined temporal trends in BWF among children hospitalized with malaria in Uganda between 2008 and 2025.

## MATERIALS AND METHODS

We analyzed data from 3,340 children hospitalized with severe malaria who were enrolled in five clinical studies conducted between 2008 and 2025 at Mulago National Teaching and Referral Hospital in central Uganda and Jinja Regional Referral Hospital in southeastern Uganda.^1^^,^^7^^–^^10^ Blackwater fever was operationally defined across studies as caregiver-reported passage of dark or cola-colored urine before admission. The presence of dark or cola-colored urine was systematically assessed from caregivers at enrollment using standardized case report forms across all five studies. Children were eligible if they were between 3 months old and 16 years old, required inpatient care, had laboratory evidence of malaria by rapid diagnostic test or microscopy, and received parenteral antimalarial therapy. Information on demographic characteristics, clinical features, and antimalarial exposure was collected using standardized case report forms.

## STATISTICAL ANALYSES

Participant characteristics were summarized overall and by study cohort to assess differences in demographic characteristics, clinical presentation, and reported prehospital medication exposure across the study period. Continuous variables are presented as medians (interquartile ranges), and categorical variables are presented as numbers (percentages).

Multivariable logistic regression was used to estimate the association between calendar year and BWF, with calendar year modeled as a continuous variable. The model adjusted for study cohort; site; age; sex; coma; severe anemia; acidotic breathing; jaundice; vomiting; diarrhea; and reported exposure to quinine, artesunate, artemether–lumefantrine, and antibiotics. Missing values were imputed using multiple imputation by the chained equations algorithm (*m =* 100 imputations, package mice in R, R Foundation, Vienna, Austria),^11^ and model estimates across imputations were pooled using the Rubin rules. For graphical representation, a best-fit curve was generated by calculating the marginally standardized predicted probabilities of BWF by calendar year from the multivariable model. Statistical significance was assessed using two-sided *P*-values.

## RESULTS

Participant characteristics differed across the five study cohorts (Table 1).^12^ Median age ranged from 1.5 years old in study 3 to 4.6 years old in study 5, and the prevalences of severe malaria manifestations varied substantially: coma (6.5%–59%), acidotic breathing (5.9%–48%), severe anemia (16%–58%), and jaundice (11.5%–60.3%). Reported prehospital antimalarial exposure also changed across studies, with declining quinine exposure and increasing prereferral artesunate exposure.

**Table 1:** Description of study cohorts

Study	Combined Data	Study 1	Study 2	Study 3	Study 4	Study 5
Years	2008–2025	2008–2013	2011–2013	2012–2013	2014–2017	2023–2025
Sample size	3340	501	178	1,572	600	489
Site						
Mulago NRH (Kampala)	1,082 (32)	501 (100)	0	0	332 (55)	249 (51)
Jinja RRH	2,258 (68)	0	178 (100)	1,572 (100)	268 (45)	240 (49)
Age, years, median (IQR)	2.0 (1.2–3.3)	3.3 (2.2–4.6)	2.0 (1.0–3.0)	1.5 (0.8–2.3)	2.0 (1.4–2.8)	4.6 (2.8–7.4)
Female sex, *n* (%)	1,892 (57)	299 (60)	101 (57)	857 (55)	337 (56)	298 (61)
Severe malaria complications						
Prostration, *n* (%)	1,712 (51)	375 (75)	162 (91)	404 (26)	428 (72)	343 (70)
Seizures, *n* (%)	1,149 (34)	254 (51)	142 (80)	306 (19)	302 (51)	145 (30)
Coma, *n* (%)	580 (18)	266 (53)	105 (59)	99 (6.5)	73 (12.3)	37 (7.6)
Acidotic breathing, *n* (%)	647 (19)	40 (8.0)	86 (48)	364 (23)	128 (21)	29 (5.9)
Elevated lactate, >5 mmol/L, *n* (%)	1,026 (31)	192 (38)	59 (33)	421 (27)	229 (38)	125 (26)
Delayed capillary refill time, >2 seconds, *n* (%)	130 (4.1)	2 (0.5)	10 (5.6)	84 (5.5)	33 (5.6)	1 (0.2)
Severe anemia, Hb <5 g/dL, *n* (%)	1,062 (32)	290 (58)	29 (16)	384 (24)	242 (41)	117 (24)
Jaundice, *n* (%)	848 (25.4)	302 (60.3)	28 (15.7)	180 (11.5)	137 (23.0)	201 (41.1)
Diarrhea, *n* (%)	722 (21.7)	32 (6.4)	38 (21.5)	422 (26.8)	114 (19.1)	116 (23.7)
Vomiting, *n* (%)	1,464 (43.9)	216 (43.1)	72 (40.5)	513 (32.7)	316 (53.0)	347 (71.0)
AKI,* *n*/*N* (%)	1,050/2,470 (42.5)	167/479 (34.9)	57/175 (32.6)	389/735 (52.9)	230/595 (38.7)	207/486 (42.6)
Stage 3 AKI, *n*/*N* (%)*	211/2,470 (8.5)	23/479 (4.8)	15/175 (8.6)	25/735 (3.4)	51/595 (8.6)	97/486 (20.0)
Reported prior drug exposure						
Any antibiotic	1,002 (30)	99 (20)	73 (41)	464 (30)	202 (34)	164 (34)
Any antimalarial	1,702 (51)	288 (57)	104 (58)	688 (44)	350 (59)	272 (56)
Artemether–lumefantrine	472 (14)	87 (17)	38 (21)	NA	212 (36)	135 (28)
Quinine	395 (12)	224 (45)	54 (30)	NA	89 (15)	28 (5.7)
Artesunate	312 (9.4)	50 (10)	0 (0)	NA	90 (15)	172 (35)
Outcome						
Died, *n* (%)	174 (5.2)	35 (7.0)	15 (8.4)	64 (4.1)	43 (7.2)	17 (3.5)

BWF status was available for 3,336 of 3,340 enrolled participants. Data are presented as *n* (percentage) unless otherwise indicated. Percentages are calculated among participants with available data; missingness was <1% unless otherwise indicated. AKI = acute kidney injury; Hb = hemoglobin; IQR = interquartile range; NA = not assessed; NRH = national referral hospital; RRH = regional referral hospital.

* AKI was defined and staged on enrolment using the Kidney Disease: Improving Global Outcomes criteria based on a 1.5× increase in creatinine from estimated baseline. Children with a creatinine value <0.4 mg/dL were not considered to have AKI.^12^

Despite these differences in participant characteristics across study cohorts, there was a marked increase in the frequency of BWF at admission from 24 of 150 (16%) before 2010 to 259 of 489 (53%) after 2020 (Figure 1). Calendar year remained associated with BWF after adjustment for study cohort; site; age; sex; coma; severe anemia; acidotic breathing; jaundice; vomiting; diarrhea; reported exposure to quinine, artesunate, and artemether–lumefantrine; and reported antibiotic exposure; the year-over-year odds ratio was 1.18 (95% CI, 1.03–1.34; *P* = 0.010), corresponding to adjusted predicted probabilities of 8.6% in 2008 and 51.0% in 2025.

**Figure 1.**
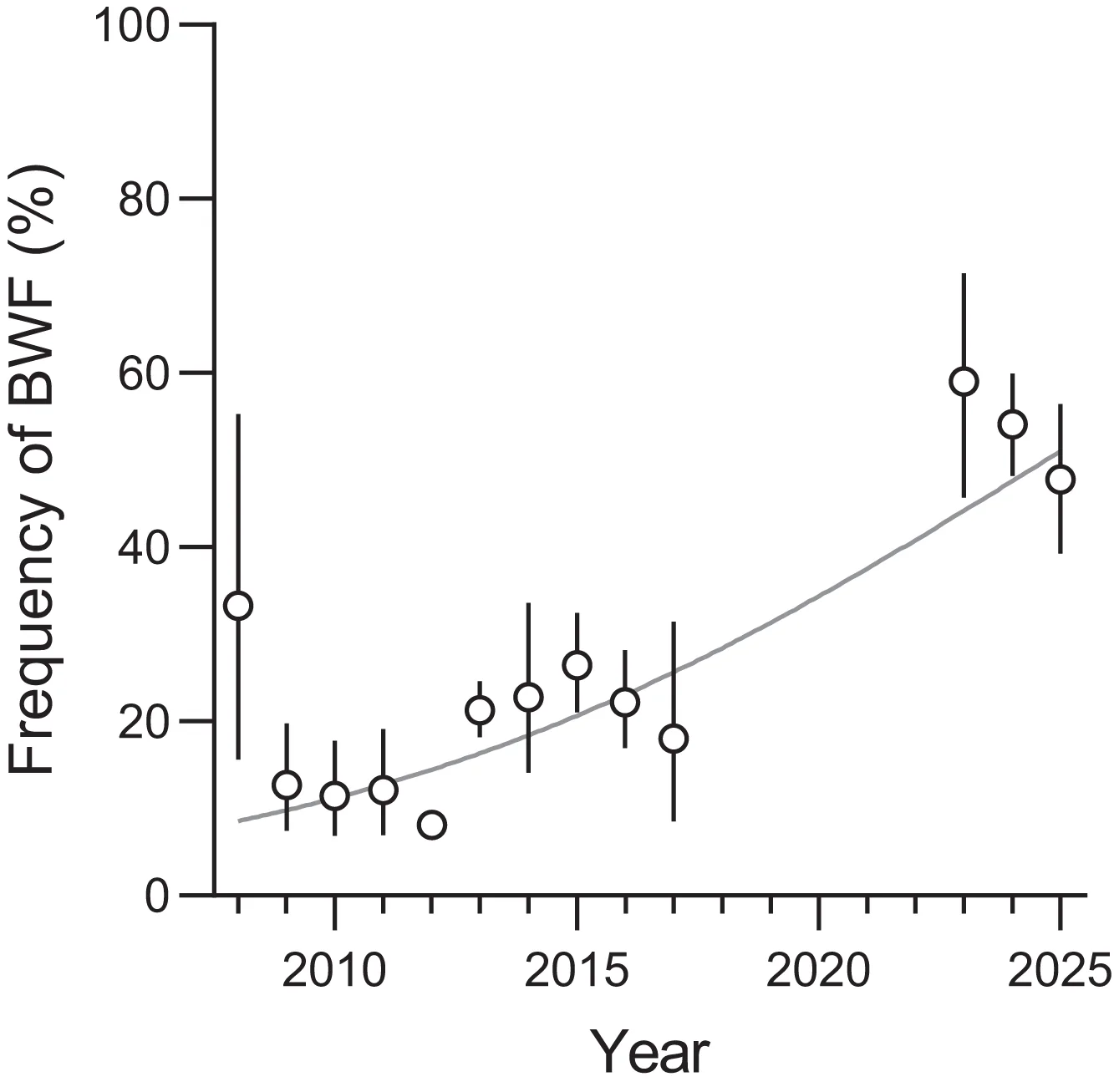
Increasing frequency of blackwater fever (BWF) among Ugandan children hospitalized with severe malaria from 2008 to 2025. Points represent annual proportions with binomial 95% CIs. The solid curve shows the marginally standardized predicted probabilities from a multivariable logistic regression model adjusting for study cohort; site; age; sex; coma; severe anemia; acidotic breathing; jaundice; vomiting; diarrhea; exposure to quinine, artesunate, or artemether–lumefantrine; and reported antibiotic exposure.

The prevalence of AKI did not increase consistently across study cohorts; however, stage 3 AKI was more frequent in the most recent cohort (20.0%) than in earlier cohorts with available creatinine measurements (3.4%–8.6%) (Table 1).^12^ Jaundice also varied substantially across cohorts, but the temporal pattern was not the same as for BWF.

## DISCUSSION

In the present analysis, we report a marked increase in BWF frequency among Ugandan children hospitalized with malaria over a 17-year period, extending beyond previously recognized high-incidence regions. Importantly, the association between calendar year and BWF persisted after adjustment for substantial differences in study cohort, site, demographic characteristics, clinical presentation, and reported prehospital medication exposure. These findings support BWF as an emerging complication of pediatric severe malaria and raise important questions regarding its epidemiology, pathogenesis, recurrence, and kidney-related morbidity.

The reasons for the observed increase in BWF remain uncertain. Changes in clinical recognition and caregiver awareness may have contributed to increased ascertainment over time. Treatment practices also changed substantially, including declining quinine exposure and increasing use of artemisinin derivatives. Adjustment for reported prehospital quinine, artesunate, and artemether–lumefantrine exposure attenuated the association between calendar year and BWF, although the temporal association remained statistically significant. Thus, changes in antimalarial exposure may account for some but not all of the observed increase. Additional explanations may include changes in parasite characteristics, environmental exposures, or other incompletely characterized mechanisms that contribute to dark urine syndromes in severe malaria.

In both historic and contemporary descriptions of severe malaria, BWF is associated with increased risk of renal failure or AKI as well as severe anemia.^9^^,^^13^ In the present cohorts, AKI prevalence did not increase consistently over time; however, stage 3 AKI was more frequent in the most recent cohort (20.0%) than in earlier cohorts with available creatinine measurements (3.4%–8.6%). More recently, BWF has been identified as a risk factor for persistent kidney disease in survivors.^1^ Although recent reports suggest that BWF is not associated with high mortality during hospitalization,^7^^,^^14^ contemporary BWF is associated with high postdischarge morbidity and mortality.^13^^,^^15^ Children with BWF frequently require blood transfusion, and recurrent episodes may further increase transfusion requirements, placing substantial pressure on blood supplies in resource-limited health systems.^16^

This analysis has limitations. Blackwater fever was operationally defined by caregiver-reported dark or cola-colored urine before admission and was not uniformly confirmed by urinalysis or measurement of urine hemoglobin. Although dark urine was systematically captured across studies, increasing clinical or community awareness of BWF over time may have increased recognition and reporting. We were unable to assess changes in the intensity or duration of urine discoloration. Although the temporal association remained significant after adjustment for reported prehospital quinine, artesunate, and artemether–lumefantrine exposure, the study was not specifically designed or powered to establish or exclude effects of individual antimalarial therapies on BWF risk. Furthermore, prehospital medication exposure was based on caregiver report and may be subject to substantial misclassification.^17^ Residual confounding by changes in treatment practices or other unmeasured differences among cohorts cannot be excluded. Finally, because the analysis included children hospitalized with severe malaria, the findings reflect changes in the frequency of BWF among hospitalized children rather than population-level incidence.

## CONCLUSION

Recognition of recurrent BWF in children highlights the need for longitudinal studies that extend beyond acute malaria hospitalization. Future work should investigate the mechanisms underlying recurrent dark urine syndromes; the relationship between BWF, AKI, and persistent kidney injury; and whether postdischarge malaria chemoprevention influences subsequent BWF risk. Improved diagnostic characterization, including urinalysis and measures of hemolysis, will be important to distinguish hemoglobinuria from other causes of dark urine and to clarify the pathogenesis of contemporary pediatric BWF.

## References

[1] Namazzi R, , 2022. Acute kidney injury, persistent kidney disease, and post-discharge morbidity and mortality in severe malaria in children: A prospective cohort study. EClinicalMedicine 44: 101292. 10.1016/j.eclinm.2022.101292 PMC8850340 35198918

[2] George CRP, 2009. Blackwater fever: The rise and fall of an exotic disease. J Nephrol 22(S14): S120–S128. 20013744

[3] Namazzi R, , 2025. Circulating immune complexes and glucose-6-phosphate dehydrogenase deficiency predict recurrent blackwater fever in Ugandan children with severe malaria. J Infect Dis 232(2): 285–297. 10.1093/infdis/jiae431 PMC12349948 39208451

[4] Olupot-Olupot P, , 2017. High frequency of blackwater fever among children presenting to hospital with severe febrile illnesses in eastern Uganda. Clin Infect Dis 64(7): 939–946. 10.1093/cid/cix003 PMC5848229 28362936

[5] Kisaakye E Basuta B Eurien D Bulage L Riolexus Ario A, 2021. *Suspected Black Water Fever among Children in Manafwa District, Eastern Uganda, 2018*. Available at: https://uniph.go.ug/suspected-black-water-fever-among-children-in-manafwa-district-eastern-uganda-2018/. Accessed October 6, 2026.

[6] Asio A Masanja V Kwiringira A Migamba SM Bulage L Kadobera D Kwesiga B Ario AR Harris J, 2022. *Black Water Fever among Children in Districts of Bugisu, Bukedi, and Busoga Regions in Eastern Uganda, January 2019–July 2021*. Available at: https://www.tephinet.org/learning/fead/black-water-fever-among-children-districts-bugisu-bukedi-and-busoga-regions-eastern. Accessed June 1, 2026.

[7] Conroy AL Hawkes MT Leligdowicz A Mufumba I Starr MC Zhong K Namasopo S John CC Opoka RO Kain KC, 2022. Blackwater fever and acute kidney injury in children hospitalized with an acute febrile illness: Pathophysiology and prognostic significance. BMC Med 20(1): 221. 10.1186/s12916-022-02410-4 PMC9248152 35773743

[8] Hawkes MT, , 2015. Inhaled nitric oxide as adjunctive therapy for severe malaria: A randomized controlled trial. Malar J 14: 421. 10.1186/s12936-015-0946-2 PMC4625637 26510464

[9] Conroy AL Opoka RO Bangirana P Idro R Ssenkusu JM Datta D Hodges JS Morgan C John CC, 2019. Acute kidney injury is associated with impaired cognition and chronic kidney disease in a prospective cohort of children with severe malaria. BMC Med 17(1): 98. 10.1186/s12916-019-1332-7 PMC6528242 31109328

[10] Batte A, , 2025. Malaria associated pathogenesis of chronic kidney disease (MAP-CKD): A prospective study of children hospitalized with severe malaria. BMC Nephrol 26(1): 390. 10.1186/s12882-025-04333-7 PMC12265236 40665227

[11] van Buuren S Groothuis-Oudshoorn K, 2011. mice: Multivariate imputation by chained equations in R. J Stat Soft 45(3): 1–67.

[12] Bond C, , Malaria Associated Kidney Injury and Disease Consortium in Africa (maKID-Africa). Identification of acute kidney injury in African children hospitalized with malaria: a multinational individual participant data meta-analysis. BMC Med. 2026. doi: 10.1186/s12916-026-05072-8. PMC13682115 42482088

[13] Opoka RO Waiswa A Harriet N John CC Tumwine JK Karamagi C, 2020. Blackwater fever in Ugandan children with severe anemia is associated with poor postdischarge outcomes: A prospective cohort study. Clin Infect Dis 70(11): 2247–2254. 10.1093/cid/ciz648 PMC7245149 31300826

[14] Paasi G Ndila C Okello F Olupot-Olupot P, 2022. Predictors of prolonged hospitalisation and mortality among children admitted with blackwater fever in eastern Uganda. Trop Doct 52(1): 61–67. 10.1177/00494755211046782 34939462

[15] Connon R, , 2021. Incidence and predictors of hospital readmission in children presenting with severe anaemia in Uganda and Malawi: A secondary analysis of TRACT trial data. BMC Public Health 21(1): 1480. 10.1186/s12889-021-11481-6 PMC8323322 34325680

[16] Obeagu EI, 2025. Blood transfusion practices in Africa: Advances, barriers, and ways of overcoming the barriers—A narrative review. Ann Med Surg (Lond) 87(11): 7311–7321. 10.1097/MS9.0000000000003898 PMC12577889 41180718

[17] Carret V, , 2025. Post-artesunate delayed hemolysis in African children with severe malaria: Incidence, medical impact and prevention. Clin Infect Dis 81(2): 389–396. 10.1093/cid/ciaf067 39964912

